# Molecularly Imprinted Polymers Based Electrochemical Sensor for 2,4-Dichlorophenol Determination

**DOI:** 10.3390/polym8080309

**Published:** 2016-08-18

**Authors:** Benzhi Liu, Hui Cang, Jianxiang Jin

**Affiliations:** School of Environmental Science and Engineering, Yancheng Institute of Technology, 224051 Yancheng, China; canghui@ycit.edu.cn (H.C.); ygjjx2000@163.com (J.J.)

**Keywords:** 2,4-dichlorophenol, electrochemical sensor, molecularly imprinted polymers

## Abstract

A molecularly imprinted polymers based electrochemical sensor was fabricated by electropolymerizing pyrrole on a Fe_3_O_4_ nanoparticle modified glassy carbon electrode. The sensor showed highly catalytic ability for the oxidation of 2,4-dichlorophenol (2,4-DCP). Square wave voltammetry was used for the determination of 2,4-DCP. The oxidation peak currents were proportional to the concentrations of 2,4-DCP in the range of 0.04 to 2.0 µM, with a detection limit of 0.01 µM. The proposed sensor was successfully applied for the determination of 2,4-DCP in water samples giving satisfactory recoveries.

## 1. Introduction

The chemical 2,4-dichlorophenol (2,4-DCP) is representative of chlorophenol compounds. It is widely used in the manufacture of some phenoxy herbicides, insecticides, and pharmaceuticals but poses remarkable environmental risks to human health due to its high toxicity, persistence in the environment, and suspected carcinogenic properties [1]. As a consequence, the US Environmental Protection Agency and European Union have listed it as a priority pollutant. Thus, the development of sensitive, simple and accurate analytical methods is required for the determination of 2,4-DCP. Many analytical methods including high performance liquid chromatography [2], gas chromatography [3], chemiluminescence [4], and electrochemical methods [5,6,7,8] have been developed to detect 2,4-DCP. Among them, electrochemical methods have some advantages for their high sensitivity, simple operation, rapid response, and small size that afford a portable sensor for on-site detection.

Recently, molecularly imprinted polymers (MIPs) based electrochemical sensors have received considerable attention due to their high selectivity and sensitivity [9,10,11]. In electrochemical sensors, MIPs can not only accumulate template molecules on the electrode surface to enhance the sensitivity, but also separate template molecules from the other analytes to improve the selectivity. For the preparation of MIPs, electropolymerization is a simple method which can directly prepare rigid, uniform, and compact MIPs film on the electrode surface [12]. Moreover, MIPs film prepared by electropolymerization has high stability, electrocatalytic activity, and conductivity, which could improve the sensitivity and selectivity of sensors. However, fewer imprinted sites formed on the electrode surface due to the relatively high density of electropolymers [13]. Because of the large surface area, nanomaterial could also be used as a carrier in the preparation of MIPs to increase the number of imprinted cavities. In this work, Fe_3_O_4_ nanoparticles were prepared and immobilized on the surface of an electrode. The polymers could be electropolymerized on the surface of Fe_3_O_4_ nanoparticles. In recent years, Fe_3_O_4_ nanoparticles have attracted much interest in the fields of separation science, electrochemistry, and catalysis, etc. [14,15,16]. Because of the large surface area and catalytic performance of Fe_3_O_4_ nanoparticles, the number of imprinted cavities could be enhanced and the selectivity and sensitivity of the sensor could be improved.

As an electroactive functional monomer, pyrrole is often employed to fabricate MIPs sensors for recognition and detection of a variety of molecules [17,18,19,20]. In this work, a simple and efficient MIPs based electrochemical sensor was prepared by electropolymerization of pyrrole on a Fe_3_O_4_ nanoparticle modified glassy carbon electrode. The sensor showed high selectivity and sensitivity for the detection of 2,4-DCP.In addition, the proposed sensor has a wide linear range and a low detection limit, which makes it suitable for the determination of trace 2,4-DCP. Recovery experiments suggest promising applicability of the sensor for the direct determination of 2,4-DCP in real samples.

## 2. Materials and Methods

### 2.1. Instrumentation and Reagents

All electrochemical experiments were carried out on a CS350 Electrochemical Workstation (Wuhan Corrtest Instruments Co., Ltd., Wuhan, China). A conventional three-electrode cell configuration was employed for the electrochemical measurements. A modified glassy carbon electrode (disc diameter of 3 mm) was used as the working electrode. The saturated calomel electrode (Saturated KCl) and platinum wire were employed as the reference and the counter electrode, respectively. Scanning electron microscopy (SEM) images were obtained using S-3400N II (Hitachi, Tokyo, Japan).

Pyrrole, 2-chlorophenol, 2,4-dichlorophenol, 2,4,6-trichlorophenol, hydroquinol, and hydroxyphenol were purchased from Sinopharm Chemical Reagent Co., Ltd. (Beijing, China). All other chemical reagents (AR grade) were obtained from Nanjing Chemical Reagent Company (Nanjing, China). Stock solution of 5.0 × 10^−4^ mol·L^−1^ 2,4-DCP was prepared by dissolving 2,4-DCP in ethanol, and then diluting to working solution at the desired concentration.

### 2.2. Fabrication of the Modified Electrodes

Fe_3_O_4_ nanoparticles were synthesized according to the following procedure. 0.86 g FeCl_2_·4H_2_O and 2.36 g FeCl_3_·6H_2_O were dissolved in 40 mL water. The mixture was magnetically stirred and purged with nitrogen gas, and then 5 mL aqueous ammonia was added. The reaction was kept for 1 h at 80 °C. After completion, the Fe_3_O_4_ nanoparticles were washed by deionized water until neutral. Then 0.1 g of neutral Fe_3_O_4_ nanoparticles were dispersed in 25 mL of methanol.

**Figure 1:**
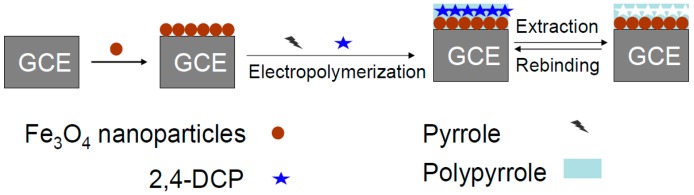
The procedure for the preparation of molecularly imprinted polymers modified glassy carbon electrode (MIPs/Fe_3_O_4_/GCE).

Subsequently, 8 μL Fe_3_O_4_ nanoparticles (4 mg·mL^−1^) were dropped onto the surface of a cleaned glassy carbon electrode (GCE) and then dried in air to prepare Fe_3_O_4_/GCE. For the preparation of MIPs/Fe_3_O_4_/GCE, the Fe_3_O_4_/GCE was incubated in a 0.1 mol·L^−1^ phosphate buffer solution (PBS) containing 6 mmol·L^−1^ pyrrole, 5 mmol·L^−1^ 2,4-DCP and 0.1 mol·L^−1^ KCl for 20 min at room temperature to complete the adsorption of 2,4-DCP and to pre-assemble between template and monomer. The electropolymerization was carried out using the cyclic voltammetry (CV) method at a scan rate of 0.1 Vs^−1^ between −0.2 and +1.2 V for 20 cycles. Then, the embedded 2,4-DCP was removed by scanning between 0 and +1.1 V in a 0.5mol L^−1^ KOH and 0.1 mol·L^−1^ KCl solution for several cycles until no obvious peak could be observed. The procedure for the preparation of MIPs/Fe_3_O_4_/GCE is depicted in Figure 1.

As a control, a non-molecularly imprinted polymers (NIPs) modified electrode (NIPs/Fe_3_O_4_/GCE) was prepared and treated in the same manner except for the addition of 2,4-DCP. A GCE was used to prepare MIPs/GCE according to the preparation of MIPs/Fe_3_O_4_/GCE.

### 2.3. Experimental Measurements

The morphology of prepared Fe_3_O_4_ nanoparticles and MIPs/Fe_3_O_4_ were observed by using scanning electron microscopy (SEM, S-3400N II). Electrochemical measurements were carried out according to the following procedure: A certain volume of 2,4-DCP stock solution and 10 mL of 0.1 mol·L^−1^ PBS (pH 6.0) were added to an electrochemical cell, and then a three electrode system was installed in it. After 120 s incubation, the cyclic voltammograms were recorded from 0.3 to 1.1V at scan rate of 0.1 Vs^−1^, the square wave voltammograms were recorded from 0.3 to 1.1 V with a step increment of 4 mV, amplitude of 25 mV, and frequency of 15 Hz.

To investigate the applicability of the proposed sensor for the determination of 2,4-DCP, local river water samples were used for the quantitative analysis. An amount of 10 mL of the water sample was transferred to the cell containing 10 mL of 0.1 mol·L^−1^ PBS (pH 6.0) and detected by square wave voltammetry under optimal conditions. The recovery experiments were performed by adding 2,4-DCP with two concentration levels and each sample was determined three times under the same conditions by square wave voltammetry.

## 3. Results and Discussion

### 3.1. Morphology of Fe_3_O_4_ Nanoparticles and MIPs/Fe_3_O_4_

**Figure 2:**
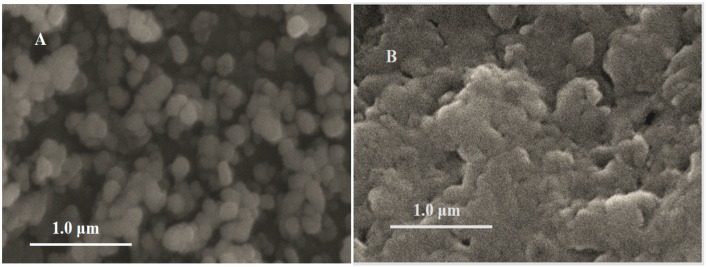

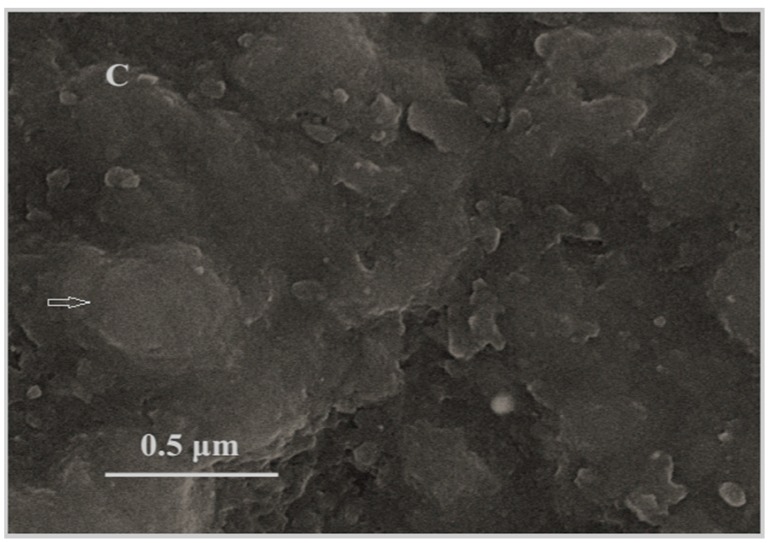
Scanning electron microscopy (SEM) images of (**A**) Fe_3_O_4_ nanoparticles; (**B**) MIPs/Fe_3_O_4_; and (**C**) high resolution of MIPs/Fe_3_O_4_.

The surface morphology of Fe_3_O_4_ nanoparticles and MIPs/Fe_3_O_4_ were evaluated by SEM. As shown in Figure 2, Fe_3_O_4_ nanoparticles were uniformly dispersed without obvious aggregation (Figure 2A), the size of Fe_3_O_4_ nanoparticles was about 120 nm. After electropolymerization, the surface became much rougher, indicating the deposition of polymers. The polymers seemed to be coated on the surface of the Fe_3_O_4_ nanoparticles (Figure 2B). As shown with the arrow in Figure 2C, a cauliflower-like polymer could be observed, but it is not obvious.

### 3.2. Electrochemical Behavior of 2,4-DCP at Modified Electrodes

**Figure 3:**
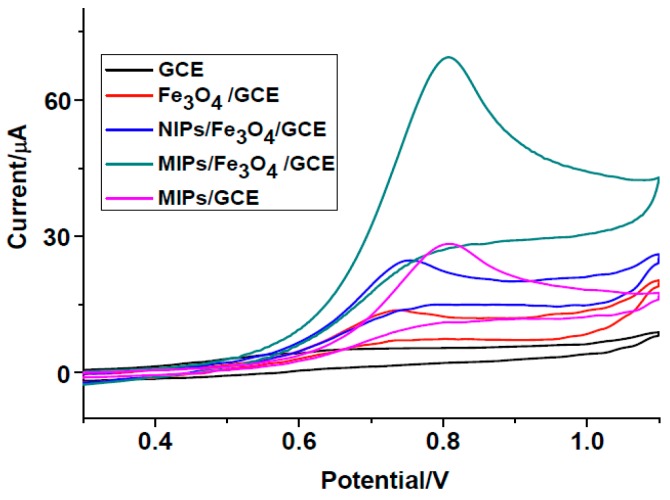
Cyclic voltammograms (CVs) of modified electrodes in 0.1 mol·L^−1^ phosphate buffer solution (PBS) containing 50 μM of 2,4-DCP. Scan rate: 0.1 Vs^−1^.

Use of cyclic voltammograms is an effective tool for studying the electrochemical properties of the modified electrodes. Figure 3 shows the CV responses of different modified electrodes in 0.1 mol·L^−1^ PBS containing 50 μM of 2,4-DCP. As can be seen, no obvious peak is found for bare GCE. A poor oxidation peak could be observed on the Fe_3_O_4_/GCE due to the weak catalysis of Fe_3_O_4_. However, there is a well-defined oxidation peak on the MIPs/GCE, indicating that pyrrole could be used to prepare electropolymers and the polymers had high catalytic ability for the oxidation of 2,4-DCP. A large well-defined oxidation peak is observed on the MIPs/Fe_3_O_4_/GCE, the peak current is about 2.8 times that of NIPs/Fe_3_O_4_/GCE, which indicated that MIPs/Fe_3_O_4_/GCE had high selectivity to the adsorption of 2,4-DCP.

### 3.3. Optimization of MIPs/Fe_3_O_4_/GCE Preparation Conditions

In order to fabricate a highly sensitive sensor, the influences of different preparation conditions including the amount of Fe_3_O_4_ nanoparticles, the ratio of template/monomer, electropolymerization scan cycles and scan rate on the response of the sensor to 20 μM of 2,4-DCP were investigated.

**Figure 4:**
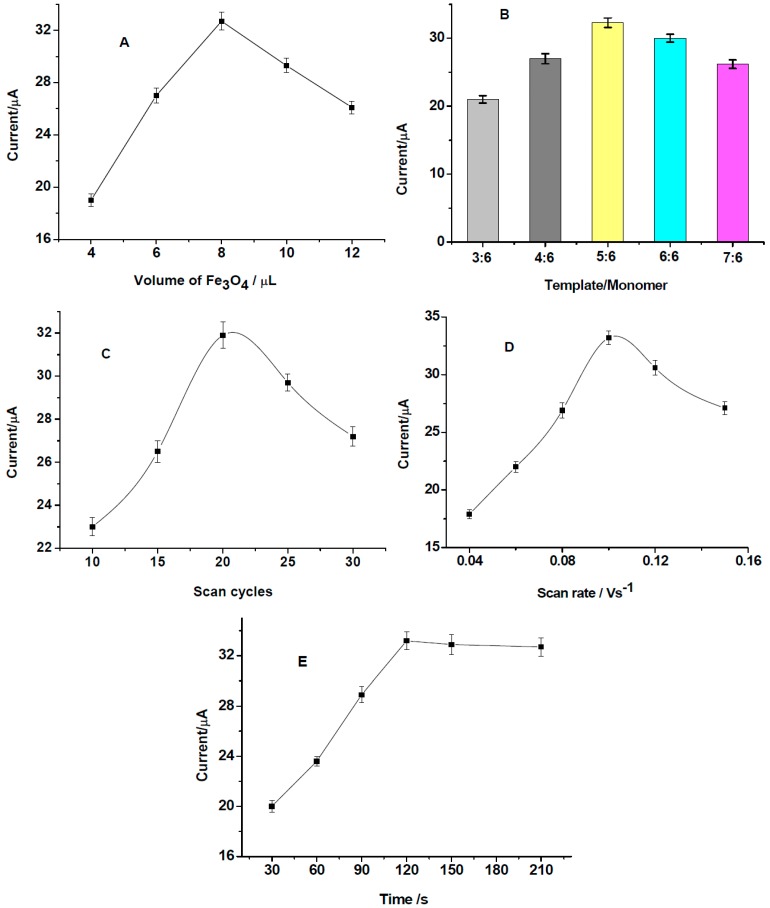
Influences of different preparation conditions on the response of the sensor to 20 μM of 2,4-DCP: (**A**) amount of Fe_3_O_4_ nanoparticles; (**B**) the ratio of template/monomer; (**C**) electropolymerization scan cycles; (**D**) scan rate; (**E**) incubation time.

In this work, Fe_3_O_4_ nanoparticles were used to enhance the immobilized amounts of imprinted cavities for adsorption of templates. It can be seen that the highest peak current was obtained for 8 μL of the prepared Fe_3_O_4_ nanoparticles (Figure 4A). In the electrodeposition of MIPs, the ratio of template/monomer could influence the amount of template molecules embedded in the polymer matrix. The results suggested that the template/monomer ratio of 5:6 exhibited the highest peak current for the sensor (Figure 4B).

The thickness of the MIPs was another important parameter that affected the sensitivity and selectivity of the sensor. Although greater deposition of templates leads to a higher number of imprinted sites, it is difficult to remove the template completely from excessively thick polymers, which lead to low binding capacity and slow kinetics [21]. Electropolymerization scan cycles and scan rates are important factors for the preparation of MIPs, which could affect the thickness and compactness of the polymers. As can be seen, the 20 cycles of scanning (Figure 4C) and scan rate of 0.1 Vs^−1^ (Figure 4D) are the optimal electropolymerization conditions. The polymers are unstable and could not coat the electrode surface completely when the scan cycles were less than 20. Higher cycles lead to the formation of thicker polymers, which also affect the sensitivity of the sensor. A slower scan rate could form tight polymers, which decrease the number of accessible imprinted sites. However, a higher scan rate could form loose and rough polymers, which could affect the stability and specificity adsorption of the polymers [22].

The incubation time of the MIPs in the analyte solution is another critical factor for the performance of the imprinted sensor. As can be seen from Figure 4E, the peak current increases with increasing incubation time from 30 to 120 s and then levels off after 120 s. Therefore, an incubation time of 120 s was selected for the following measurements.

### 3.4. Determination of 2,4-DCP

**Figure 5:**
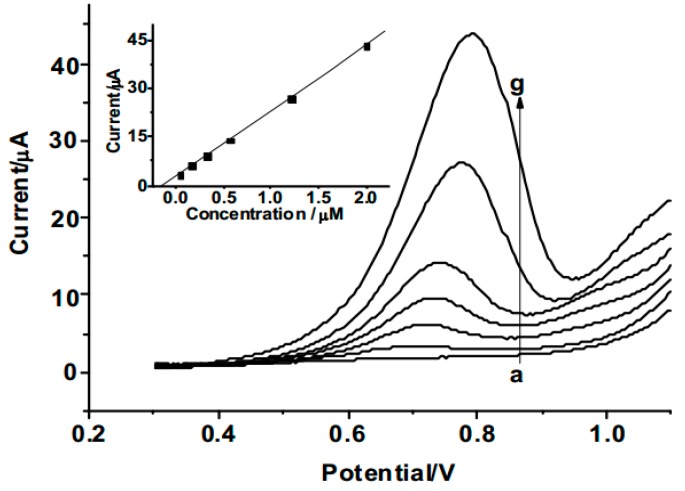
Square wave voltammetry (SWVs) of MIPs/Fe_3_O_4_/GCE in solution containing different concentrations of 2,4-DCP, from **a**–**g**: 0, 0.04, 0.16, 0.32, 0.56, 1.2, 2.0 µM. Inset: plot of peak current versus 2,4-DCP concentration.

Square wave voltammetry (SWV) was used for the determination of 2,4-DCP due to its higher current sensitivity and better resolution than cyclic voltammetry. Figure 5 shows the SWVs of MIPs/Fe_3_O_4_/GCE in electrolyte solution containing different concentrations of 2,4-DCP. The oxidation peak currents of 2,4-DCP are proportional to their concentrations in the range from 0.04 to 2.0 µM, with a detection limit of 0.01 µM (inset).According to the IUPAC recommendation [23], the detection limit is determined using 3ó/slope ratio, where ó is the standard deviation of the mean value for 10 determinations of the blank. The linear regression equation can be expressed as *I*_pa_ (µA) = 2.73 + 20.5*c* (µM), with a correlation coefficient *r* = 0.9994.

In addition, the determination performance of the sensor fabricated in this work was compared with other electrochemical methods. As shown in Table 1, it is clear that the proposed sensor has a wide linear range and a low detection limit, which makes it suitable for the determination of trace 2,4-DCP.

### 3.5. Reproducibility and Stability

The reproducibility and stability of the proposed sensor were studied. The data results were shown in Table 2. To investigate the reproducibility of the proposed sensor, a series of four sensors prepared in the same manner were tested for the determination of 0.3 µM 2,4-DCP and the RSD was 2.4%. The stability of the sensor was also studied, when the prepared sensor was stored at room temperature after two weeks, the peak current response retained 93% of its initial response.

### 3.6. Selectivity Study

**Figure 6:**
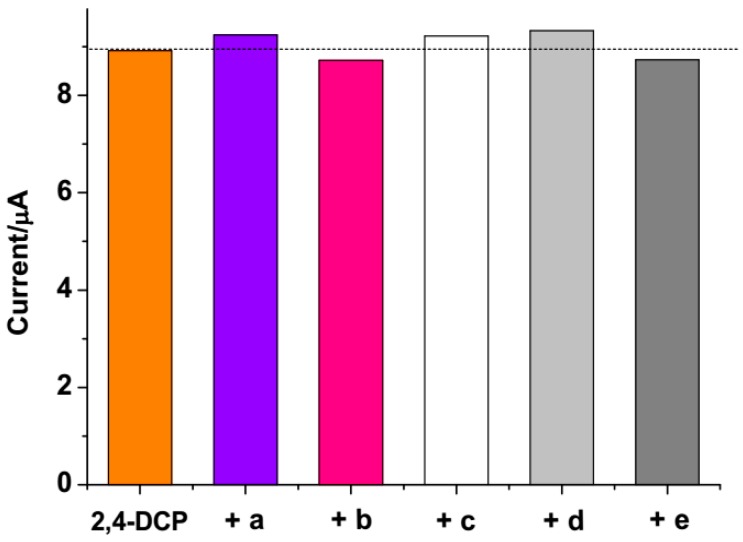
The peak current changes of 0.3 µM 2,4-DCP at MIPs/Fe_3_O_4_/GCE with addition of 5-fold concentration of interference species: (**a**) 2-chlorophenol; (**b**) hydroxyphenol; (**c**) pentachlorophenol; (**d**) 2,4,6-trichlorophenol; (**e**) hydroquinol.

To verify the selectivity of the proposed sensor, hydroquinol, hydroxyphenol, 2-chlorophenol, 2,4,6-trichlorophenol, and pentachlorophenol were selected in the interference experiments. The interference experiments were carried out by detecting the current response of 0.3 µM 2,4-DCP at MIPs/Fe_3_O_4_/GCE in the presence of a 5-fold concentration of the interference species. As can be seen in Figure 6, the above species did not show obvious interference to the 2,4-DCP detection. Moreover, the effect of several ions on the determination of 2,4-DCP was also studied. The results showed that 200-fold concentrations of Na^+^, K^+^, Zn^2+^, Mg^2+^, Al^3+^, Ca^2+^, Cl^−^, NO_3_^−^, SO_4_^2−^ have no interference on the determination of 2,4-DCP. The results suggested that the proposed sensor has good selectivity for the detection of 2,4-DCP.

### 3.7. Real Water Sample Analysis

To investigate the applicability of the proposed sensor for the determination of 2,4-DCP, local river water samples were used for the quantitative analysis. No obvious electrochemical response was found for the water samples. It is assumed that there is no 2,4-DCP in the river sample or the concentration of 2,4-DCP is too low to be detected. Thus, the recovery experiments were performed by adding known concentrations of 2,4-DCP. The data are listed in Table 3. The recoveries range from 94.2% to 97.5%, which indicate the applicability and reliability of the proposed sensor.

## 4. Conclusions

In this study, a simple and efficient MIPs based electrochemical sensor was prepared by electropolymerization of pyrrole on a Fe_3_O_4_ nanoparticle modified glassy carbon electrode. The influences of different preparation conditions including amount of Fe_3_O_4_ nanoparticles, the ratio of template/monomer, electropolymerization scan cycles and scan rate on the response of the sensor to 2,4-DCP were investigated. This has provided a technique basis for the preparation of other Fe_3_O_4_ nanoparticles based MIPs. Under the optimum preparation conditions, the sensor showed high selectivity and sensitivity, wide linear range, and low detection limit, which makes it a good sensor for the detection of 2,4-DCP. The applicability of the proposed sensor for the determination of 2,4-DCP in real water samples was performed with good recoveries. The proposed sensor represents a new platform for designing electrochemical sensors for environmental pollutants.

## Figures and Tables

**Table 1:** The determination performance comparison with other electrochemical methods.

Modified electrode	Linear range (μM)	LOD (μM)	References
Nafion/MWNTs/GCE	0.1–100	0.037	[5]
Tyrosinase/MWNTs/GCE	2.0–100	0.66	[6]
Lac/PVA/F108/Au NPs/GCE	1.0–25.0	0.04	[7]
Mb-AG/GCE	12.5–208	2.06	[24]
HRP/MWNTs/GCE	1.0–100	0.38	[25]
MIPs/Fe_3_O_4_/GCE	0.04–2.0	0.01	this work

MWNTs, multiwalled carbon nanotubues; Lac, laccase; PVA, polyvinyl alcohol; F108, polyethyleneoxide–polyoxypropylene–polyethyleneoxide (PEO–PPO–PEO); Au NPs, gold nanoparticles; MB-AG, Myoglobin and agarose; HRP, horseradish peroxidase.

**Table 2:** Data results of reproducibility and stability.

Items	Current response of sensors (μA)				RSD (%) (*n* = 4)
	Sensor 1	Sensor 2	Sensor 3	Sensor 4	
Reproducibility	8.79	9.55	8.46	9.23	2.4
	0 day	3 day	7 day	14 day	
Stability	9.11	8.98	8.72	8.47	

**Table 3:** Analysis of 2,4-DCP in spiked water samples.

River water	Added (µM)	Found (µM)	Recovery (%)	RSD (%) (*n* = 3)
	0	Not detected	–	–
Sample 1	0.16	0.153	95.6	3.9
	1.2	1.17	97.5	4.2
	0	Not detected	–	–
Sample 2	0.16	0.155	96.9	3.7
	1.2	1.13	94.2	3.4
